# Supplementary material for: Integrated polarization-sensitive amplification system for digital information transmission
Source: Nat Commun. 2021 Nov 9;12:6476. doi: 10.1038/s41467-021-26919-z (PMC8578569; doi:10.1038/s41467-021-26919-z)
Supplement: Supplementary file 1 — Supplementary information [file 41467_2021_26919_MOESM1_ESM.pdf]

Supplementary Information

Integrated polarization-sensitive amplification system for digital information transmission

Wenhao Ran[1,2], Zhihui Ren[1,2], Pan Wang[1,2], Yongxu Yan[1,2], Kai Zhao[1,2], Linlin Li[1,2], Zhexin Li[1,2], Lili Wang[1,2], Juehan Yang[1,2], Zhongming Wei[1,2]*, Zheng Lou[1,2]*, Guozhen Shen[1,2]*

[1] State Key Laboratory of Superlattices and Microstructures, Institute of Semiconductors, Chinese Academy of Sciences, Beijing 100083, China.

[2] Center of Materials Science and Optoelectronic Engineering, University of Chinese Academy of Sciences, Beijing 100049, China.

Correspondence and requests for materials should be addressed to Z.M. Wei (zmwei@semi.ac.cn), Z. Lou (zlou@semi.ac.cn) and G.Z. Shen (gzshen@semi.ac.cn).

This file includes:

Supplementary Figs 1-20
Supplementary Note 1-5
Supplementary Table 1-2
References

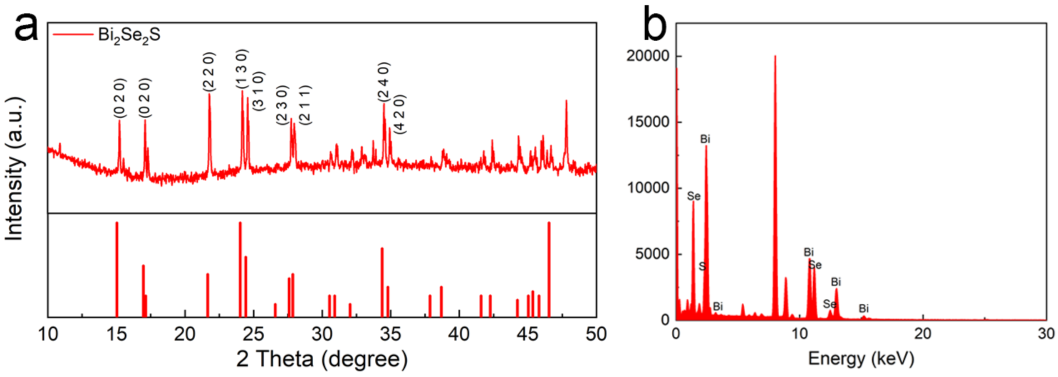

Supplementary Figure 1 a XRD image and **b** EDS image of Bi_2Se_2S NWs.

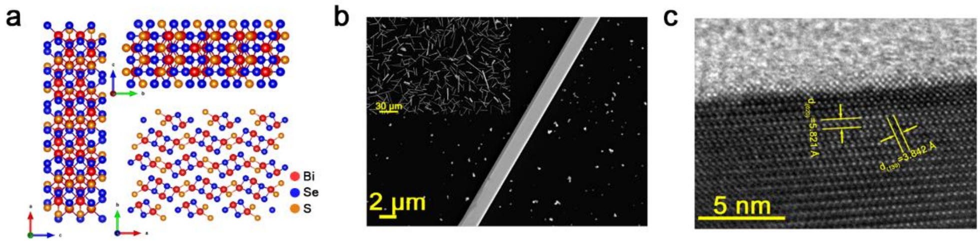

Supplementary Figure 2 a The atom structure of Bi_2Se_2S under different viewing angle. **b** The scanning electron microscopy (SEM) image of Bi_2Se_2S NWs. **c** The high-resolution transmission electron microscope (HRTEM) image of Bi_2Se_2S NWs.

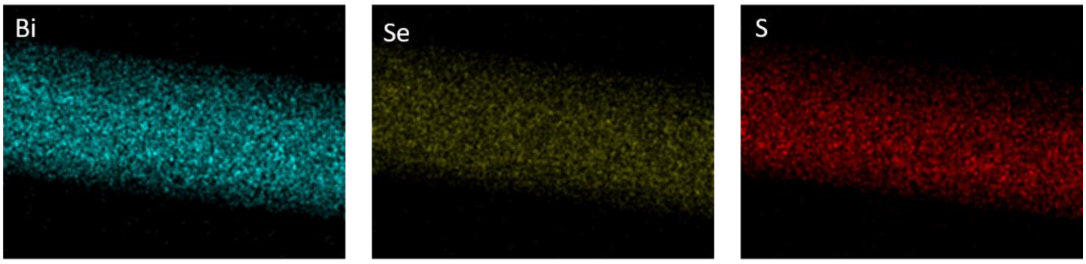

Supplementary Figure 3 EDS element mapping (Bi, Se, S) of Bi_2Se_2S NWs.

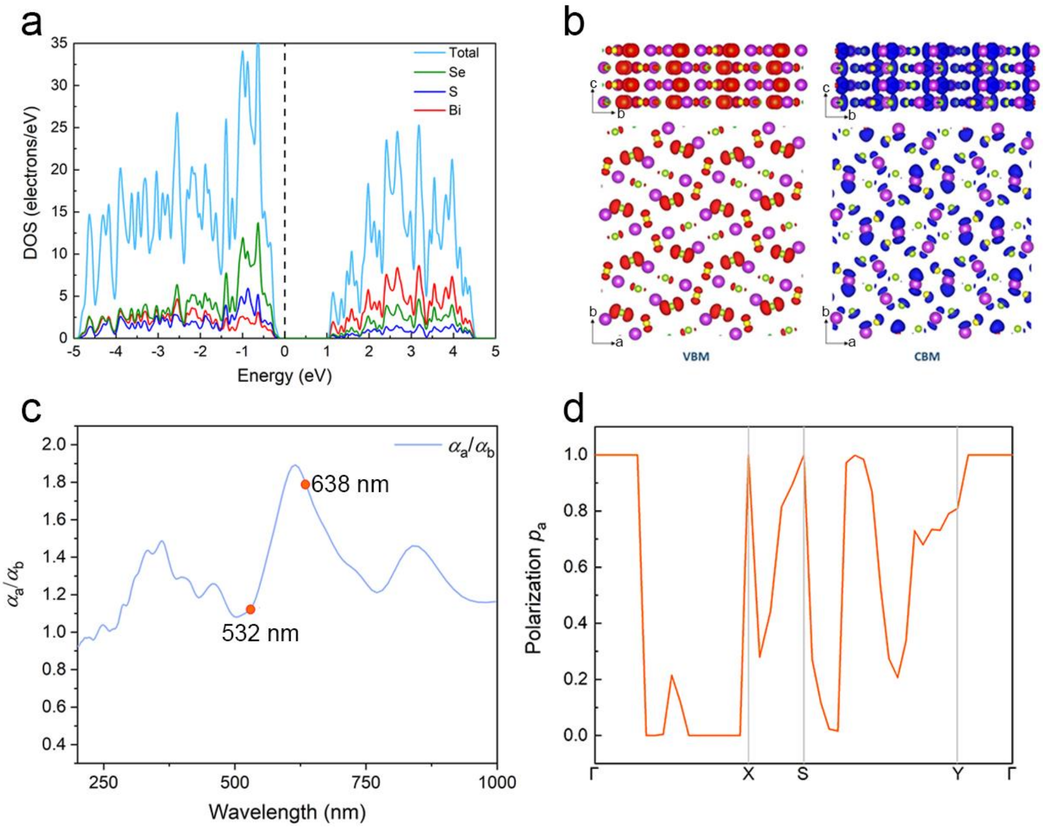

Supplementary Figure 4 a The atom-resolved and total density of states (DOS) of the bulk Bi_2Se_2S. **b** Calculated partial charge density of bulk Bi_2Se_2S at the state of VBM (left) and CBM (right). The purple, yellow and green balls represent Bi, S and Se atoms, respectively. **c** The ratio of absorption coefficient of a-axis and b-axis (α_a/α_b). **d** The p_a of optical transition from $|\varphi_{CBM}\rangle$ to $|\varphi_{VBM}\rangle$ along the k-point path.

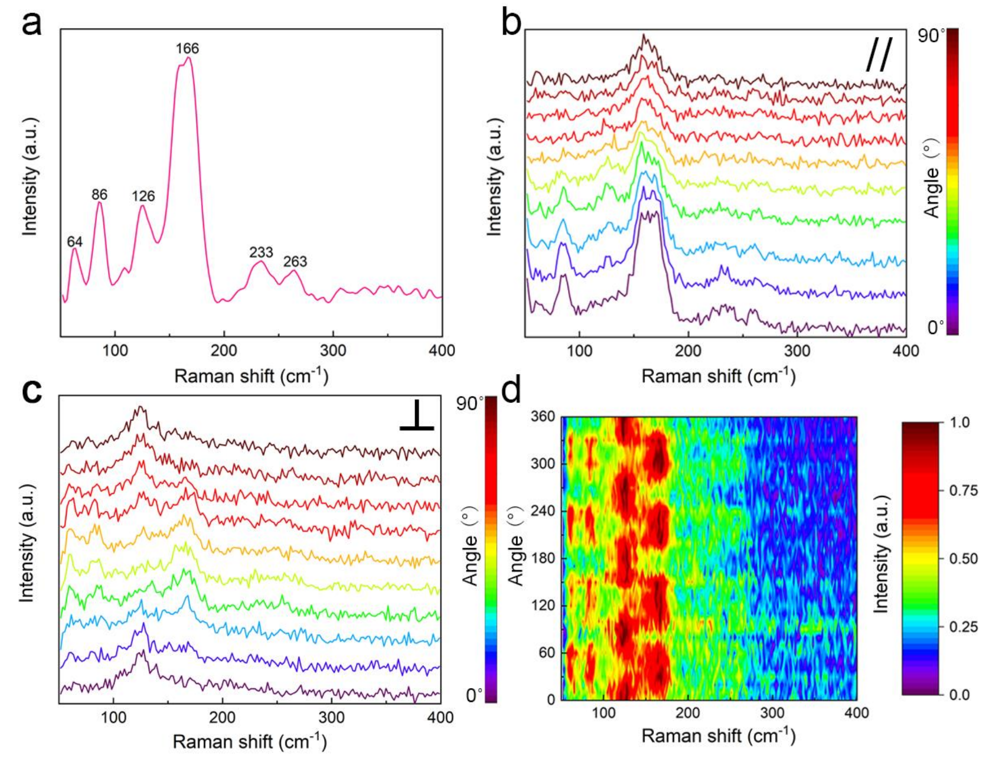

Supplementary Figure 5 a The Raman scattering spectra with unpolarized 532 nm laser. The angle-resolved Raman scattering spectra under **b** parallel configurations and **c** cross configurations of 532 nm laser. **d** The polarized Raman intensity mapping under cross configurations of 532 nm laser.

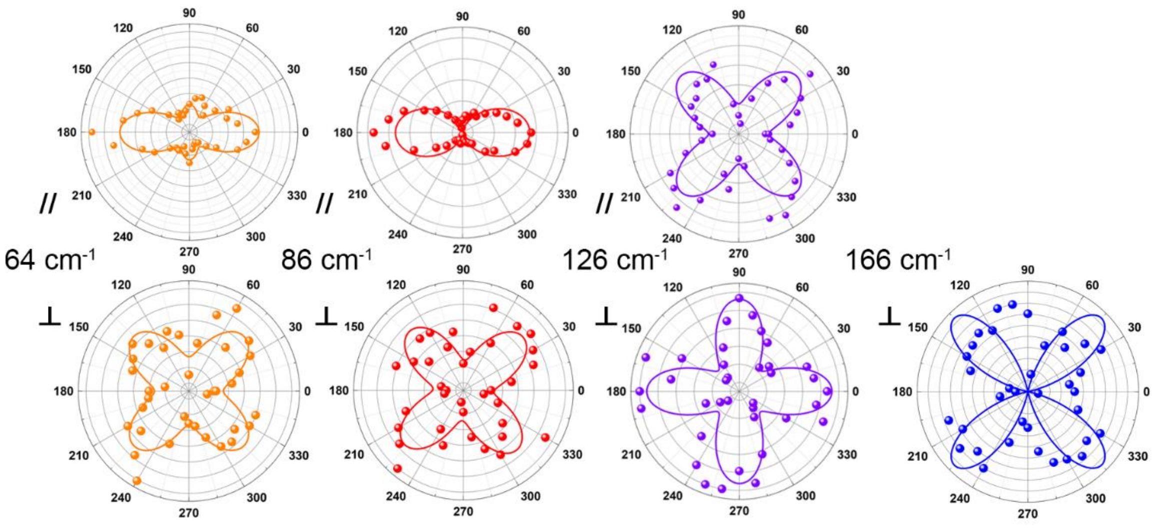

Supplementary Figure 6 The polar plot of angle-resolved Raman intensity corresponding to peak 64 cm^{-1}, 86 cm^{-1}, 166 cm^{-1} and 126 cm^{-1}, respectively under parallel configurations (//) and cross configurations (⊥). Circles and curves represent experimental data and the result of the formula fitting.

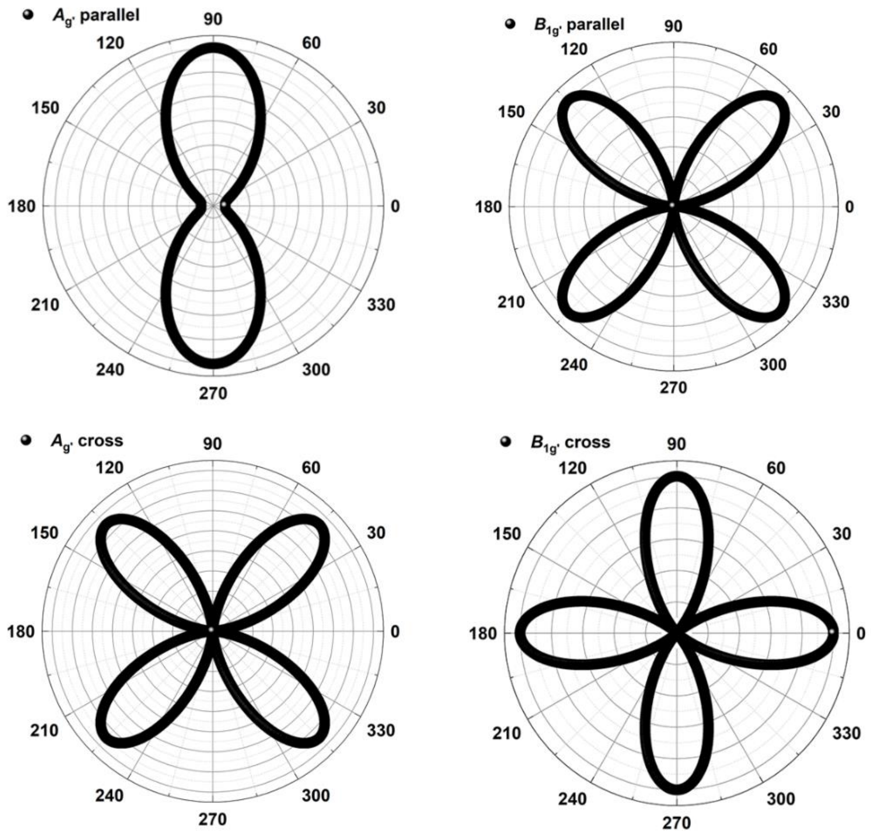

Supplementary Figure 7 The theoretical curves of A_g mode and B_{1g} mode.

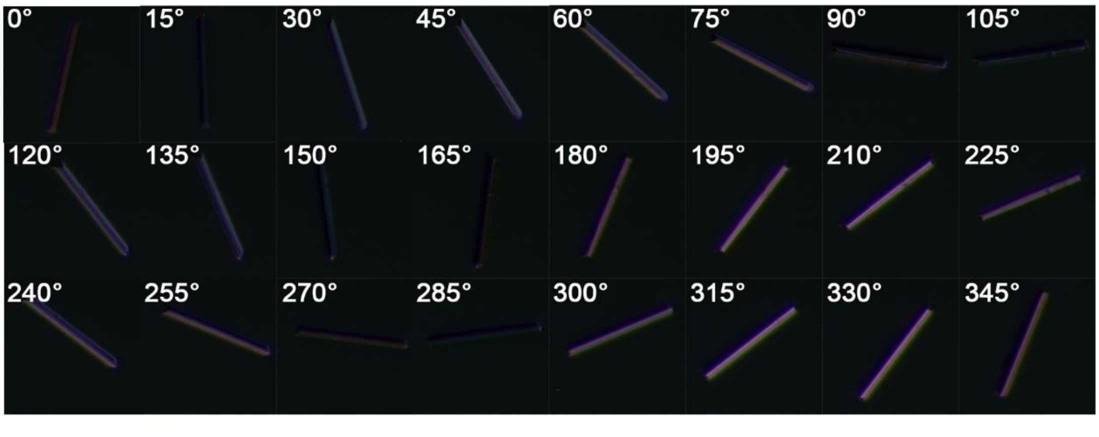

Supplementary Figure 8 PROM images of Bi_2Se_2S NWs under different angle.

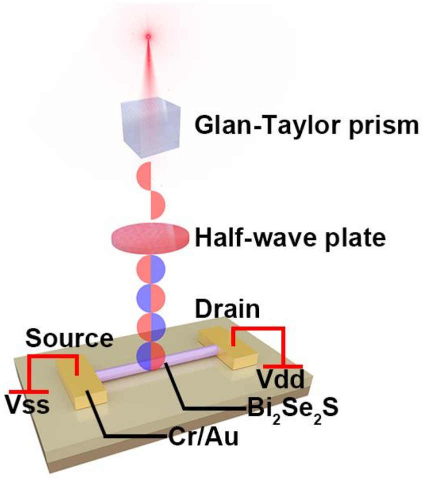

Supplementary Figure 9 Schematic diagram of Bi_2Se_2S NWs based PS structure and test system.

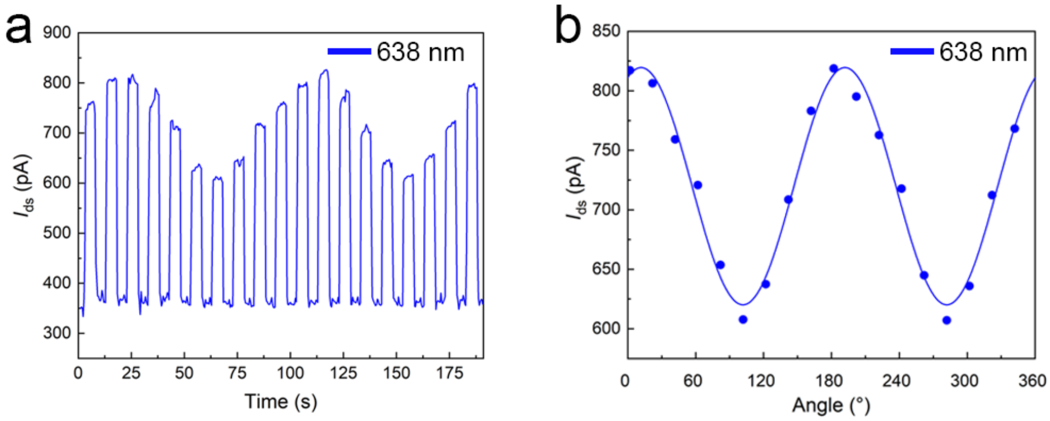

Supplementary Figure 10 a The time-resolved polarized optoelectronic response of Bi_2Se_2S NW based PS under 638 nm. **b** Rectangle coordinate diagram of relationship between photocurrent and polarization angle under 638 nm.

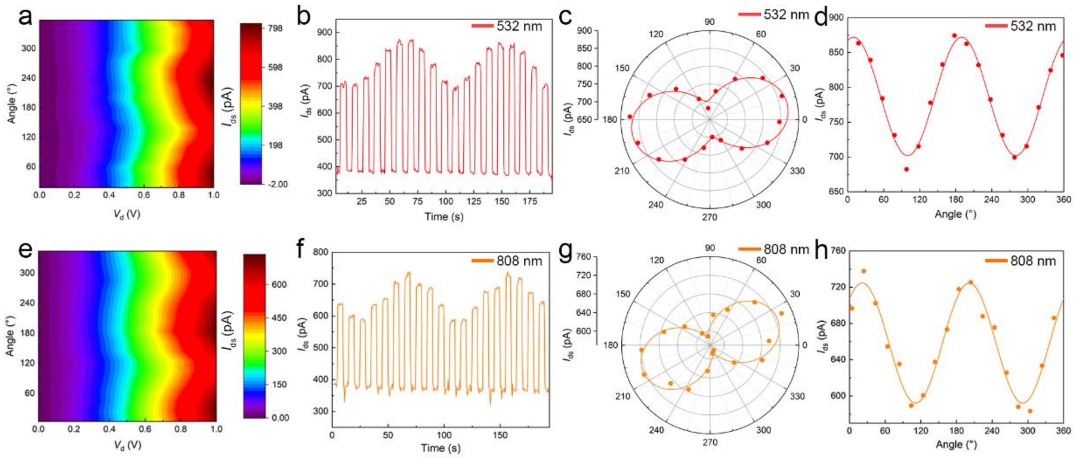

Supplementary Figure 11 Optoelectronic response of Bi_2Se_2S NW based PS. *I*-*V* curves with different polarization angle under **a** 532 nm and **e** 808 nm. Time-resolved polarized optoelectronic response under **b** 532 nm and **f** 808 nm. Polar diagram of relationship between photocurrent and polarization angle under **c** 532 nm and **g** 808 nm at 1.0 V drain bias, the anisotropic photocurrent ratio are 1.24 and 1.22. Rectangle coordinate diagram of relationship between photocurrent and polarization angle under **d** 532 nm and **h** 808 nm.

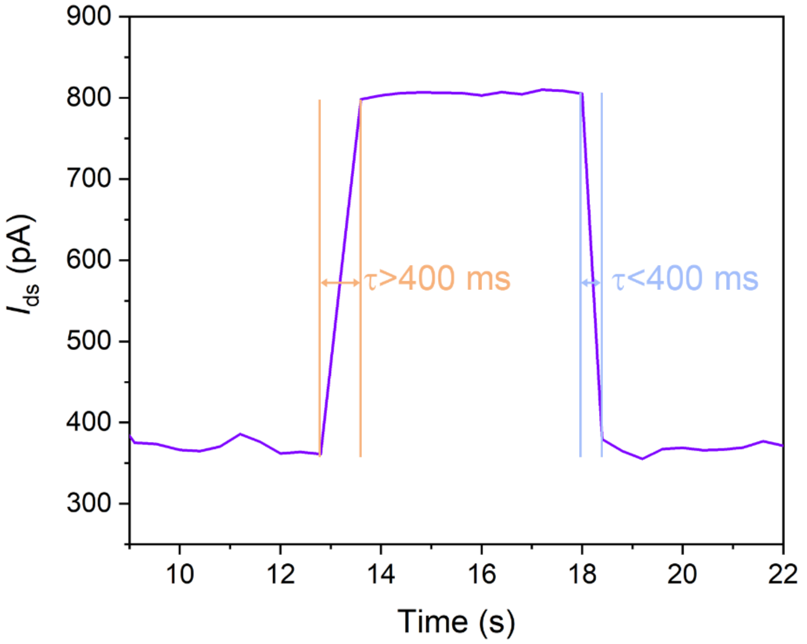

Supplementary Figure 12 The responding time of Bi_2Se_2S NW based PS.

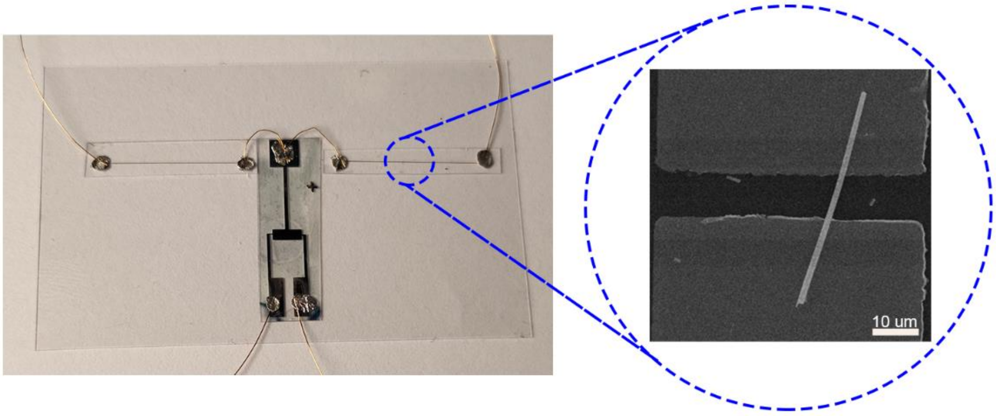

Supplementary Figure 13 The physical diagram of the PSAS.

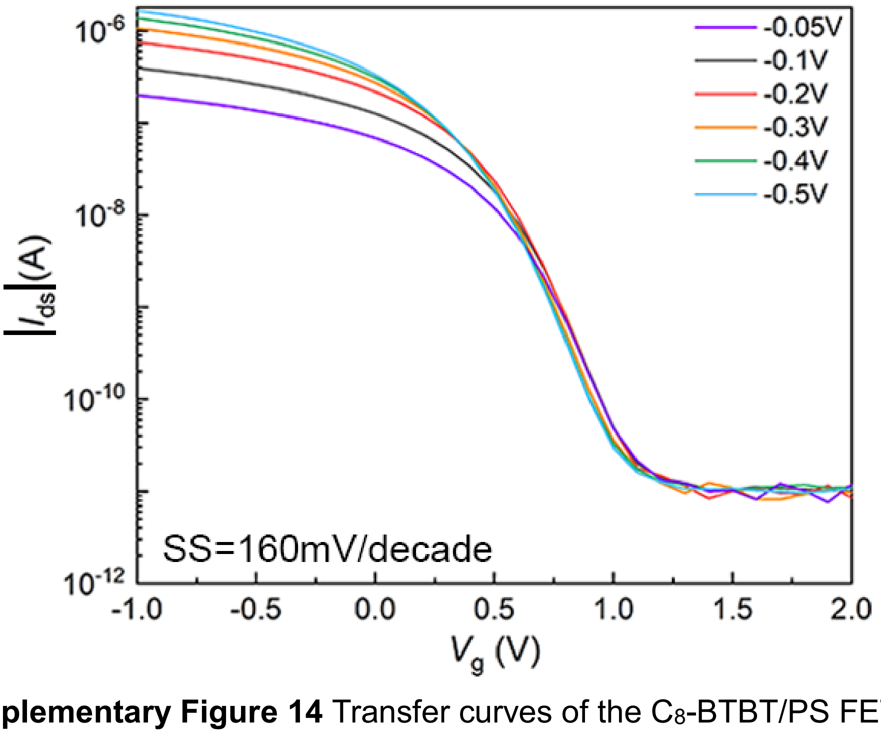

Supplementary Figure 14 Transfer curves of the C_8-BTBT/PS FET at different V_{ds}.

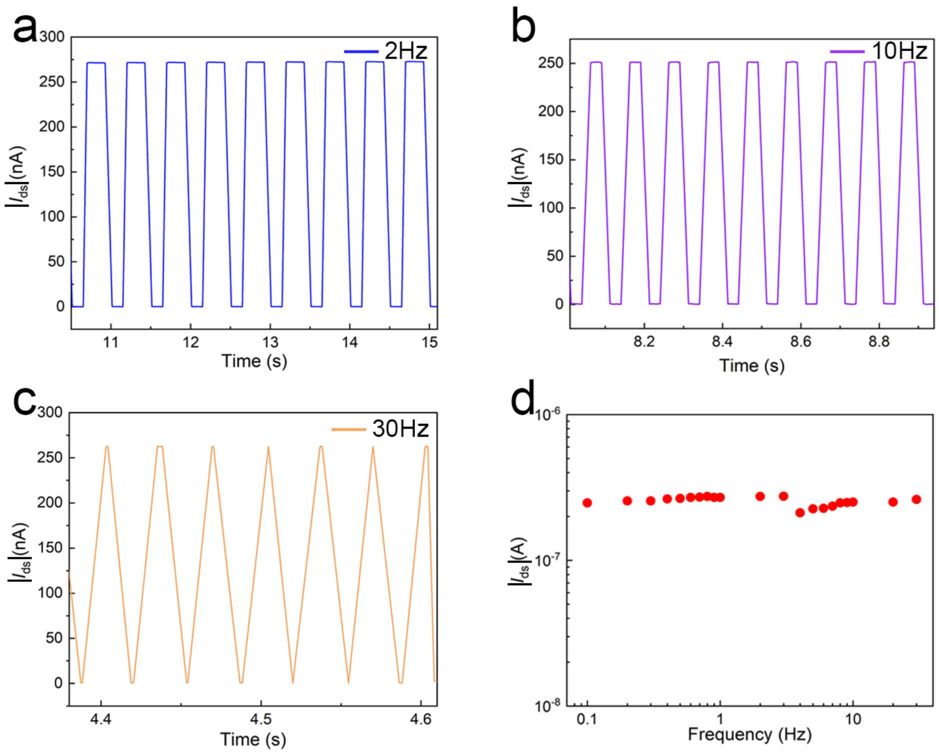

Supplementary Figure 15 Dynamic measurements of the C_8-BTBT/PS FET during continuous cycling between on/off states at **a** 2 Hz, **b** 10Hz and **c** 30 Hz under V_{ds} = -0.5 V. **d** Frequency characteristic of the C_8-BTBT/PS FET.

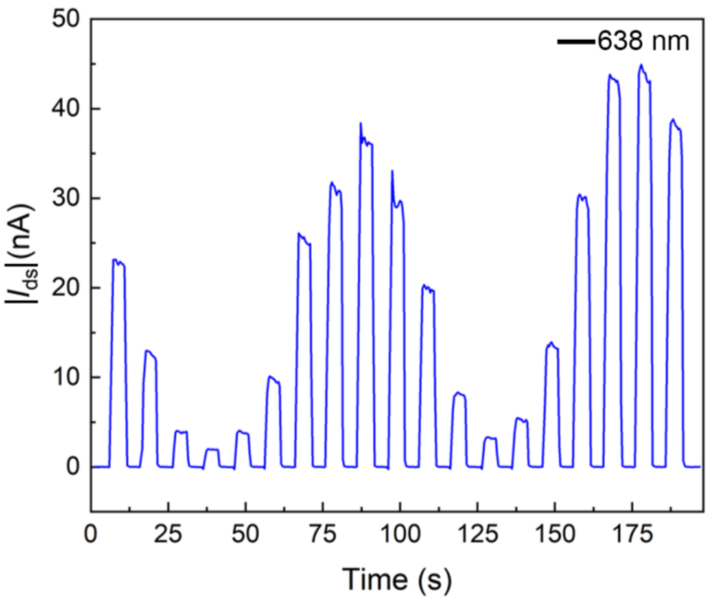

Supplementary Figure 16 The time-resolved polarized optoelectronic response of PSAS under 638 nm.

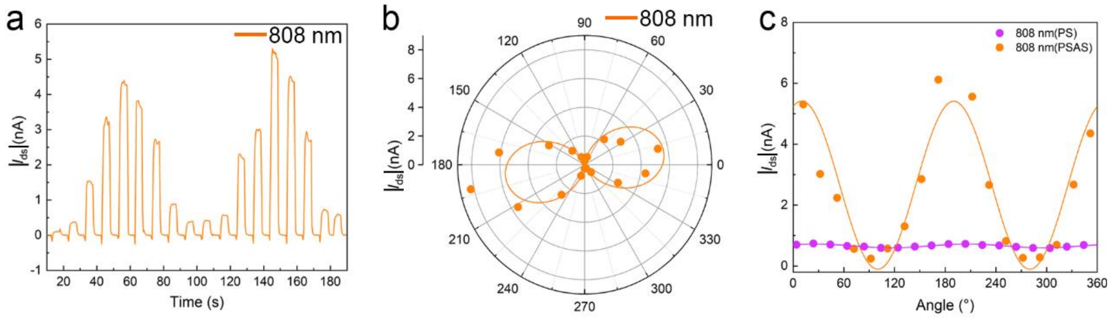

Supplementary Figure 17 Optoelectronic response of PSAS. a Time-resolved polarized optoelectronic response, **b** polar diagram of relationship between photocurrent and polarization angle and **c** rectangle coordinate diagram of relationship between photocurrent and polarization angle of PSAS and PS based Bi_2Se_2S NWs respectively under 808 nm (optical power density of 77.443 mW/cm^2).

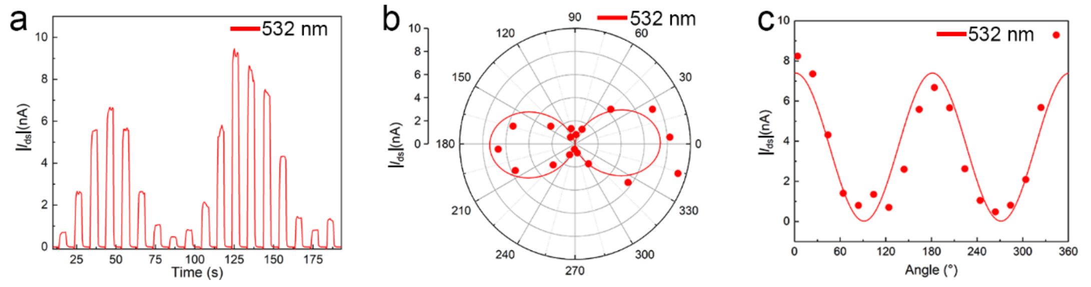

Supplementary Figure 18 Optoelectronic response of PSAS. a Time-resolved polarized optoelectronic response under 532 nm. **b** Polar diagram of relationship between photocurrent and polarization angle under 532 nm, the anisotropic photocurrent ratio is 375. **c** Rectangle coordinate diagram of relationship between photocurrent and polarization angle under 532 nm.

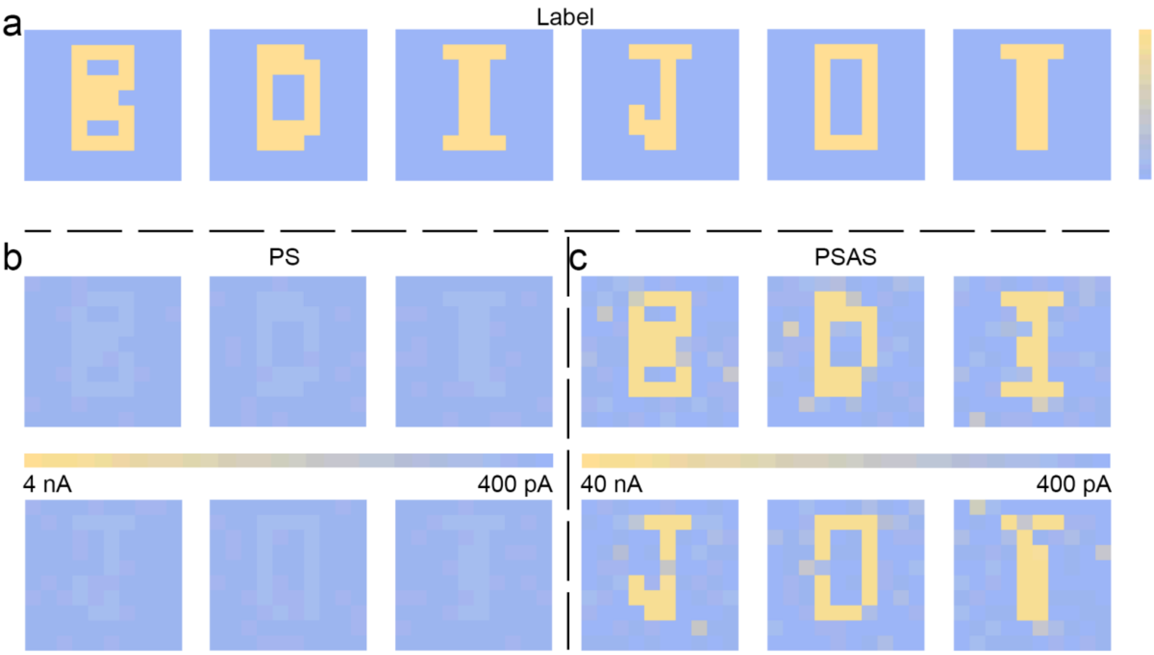

Supplementary Figure 19 a Idea patterns of six letters (B, D, I, J, O, T). **b** Incomplete pattern with background noise based on PS system. **c** Incomplete pattern with background noise based on PSAS.

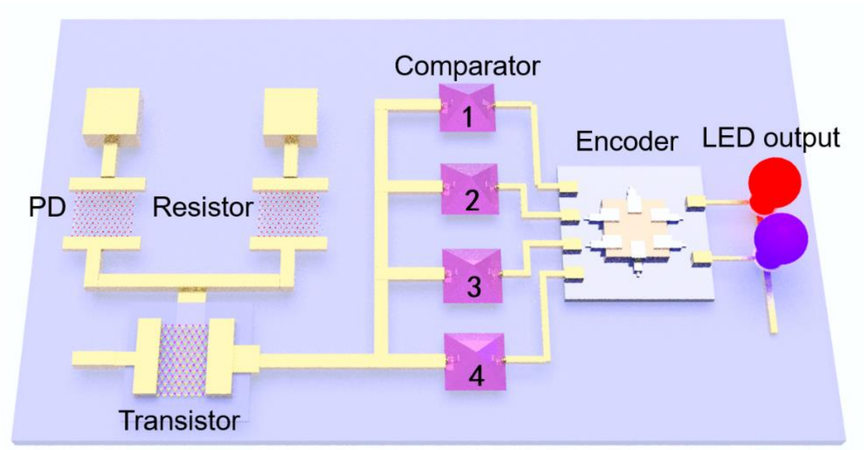

Supplementary Figure 20 The structure schematic diagram of the polarized light information decoding.

Supplementary Note 1

The Bi_2Se_2S NWs fabricated polarization-sensitive photodetector and reference resistor were synthesized by chemical vapor deposition (CVD), which X-ray diffraction (XRD) is shown in Supplementary Fig. 1a. The diffraction peaks almost identical to those of Bi_2Se_2S (JCPDS Card No. 19-0174) indicate that the NWs belong to the orthorhombic structure, which the space group is Pbnm (64). Supplementary Fig. 1b describes the energy dispersive spectrometer (EDS) image of the Bi_2Se_2S NWs, Bi: Se: S = 2:2:1 further verified that the grown NWs are Bi_2Se_2S. The crystals structure of the Bi_2Se_2S is shown in Supplementary Fig. 2a. It is evident that $(Bi_8Se_8S_4)_n$ is stacked along the a-axis through strong covalent Bi-S and Bi-Se bonds, and is held together by van der Waals forces in the b-axis. Therefore, at the edge of Bi_2Se_2S, the bonding force between atoms along the a-axis is stronger than that along the b-axis, which leading that the Bi_2Se_2S tends to grow along the a-axis. The anisotropic crystal structure makes it easier for Bi_2Se_2S to form NWs morphology during the growth process and preferentially grow along the a-axis. As shown in Supplementary Fig. 2b, the SEM image further demonstrates that the morphology of the growing material is similar to NWs and the diameter is less than 1 μm. In order to analyze the crystal structure of Bi_2Se_2S NW, HRTEM of the NW is characterized (Supplementary Fig. 2c). At this zone axis and defocus amount slightly, it can be seen that the NW has two crystal planes (0 2 0) and (1 3 0), which corresponding lattice spacing is 5.821 Å and 3.842 Å, respectively. This result further proves that the NWs preferentially grow along the a-axis. The

secondary growth axis is the b-axis. When linearly polarized light is irradiated on the surface of the material, the structure anisotropy of the a-axis and b-axis of the Bi_2Se_2S NW will be main factor affecting the anisotropic photocurrent of the material. The EDS element mapping corresponding to the TEM image is shown in Supplementary Fig. 3, demonstrating that the three elements of Bi, Se and S are uniformly distributed in the NW. This illustrates that the grown Bi_2Se_2S NWs are of high quality and crystallinity.

Supplementary Note 2

According to the classical theory, the Raman scattering intensity of the crystal can be expressed as:

$$I \propto |h_s^{\mathrm{T}} \cdot \mathbf{R} \cdot h_i|^2 \qquad (1)$$

where *I* is the Raman scattering intensity, h_s and h_i are the polarization direction vector of the incident laser and scattered laser, respectively, *R* is the Raman tensor. According to the previous inference, since the Bi_2Se_2S NWs grow along the a-b plane and stack along the c-axis, the incident light is irradiated to the a-b plane of the Bi_2Se_2S NWs along the c-axis. Therefore, the corresponding h_s and h_i can be defined as:

$$h_i = \begin{pmatrix} \cos\theta \\ \sin\theta \\ 0 \end{pmatrix} \; h_{s\parallel} = \begin{pmatrix} \cos\theta \\ \sin\theta \\ 0 \end{pmatrix} \; h_{s\perp} = \begin{pmatrix} -\sin\theta \\ \cos\theta \\ 0 \end{pmatrix} \qquad (2)$$

where *h*_(s∥) and *h*_(s⊥) correspond to the polarization direction of scattered laser in parallel and cross configurations, respectively. θ represents the angle between the polarization direction of the incident light and the a-axis. Because

the space group of Bi_2Se_2S is Pbnm and the point group is D_{2h}^{16}, its Raman tensor are mean as:

$$\mathbf{R}(A_\mathrm{g}) = \begin{pmatrix} ae^{\mathrm{i}\cdot\varphi_\mathrm{a}} & 0 & 0 \\ 0 & be^{\mathrm{i}\cdot\varphi_\mathrm{b}} & 0 \\ 0 & 0 & ce^{\mathrm{i}\cdot\varphi_\mathrm{c}} \end{pmatrix} \mathbf{R}(B_{1\mathrm{g}}) = \begin{pmatrix} 0 & de^{\mathrm{i}\cdot\varphi_\mathrm{d}} & 0 \\ de^{\mathrm{i}\cdot\varphi_\mathrm{d}} & 0 & 0 \\ 0 & 0 & 0 \end{pmatrix}$$

$$\mathbf{R}(B_{2\mathrm{g}}) = \begin{pmatrix} 0 & 0 & ee^{\mathrm{i}\cdot\varphi_\mathrm{e}} \\ 0 & 0 & 0 \\ ee^{\mathrm{i}\cdot\varphi_\mathrm{e}} & 0 & 0 \end{pmatrix} \mathbf{R}(B_{1\mathrm{g}}) = \begin{pmatrix} 0 & 0 & 0 \\ 0 & 0 & fe^{\mathrm{i}\cdot\varphi_\mathrm{f}} \\ 0 & fe^{\mathrm{i}\cdot\varphi_\mathrm{f}} & 0 \end{pmatrix} \quad (3)$$

Based on the above series of formulas, we can calculate the Raman scattering intensity corresponding to different Raman mode, which results are as follows:

$$I(A_{\mathrm{g}\parallel}) \propto \left(a\cdot\cos^2(\theta)\cdot\cos(\varphi_\mathrm{a}) + b\cdot\sin^2(\theta)\cdot\cos(\varphi_\mathrm{b})\right)^2 + \left(a\cdot\cos^2(\theta)\cdot\sin(\varphi_\mathrm{a}) + b\cdot\sin^2(\theta)\cdot\sin(\varphi_\mathrm{b})\right)^2 \quad (4)$$

$$I(A_{\mathrm{g}\perp}) \propto (b^2 + a^2 - 2\cdot a\cdot b\cdot\cos(\varphi_\mathrm{ab}))\cdot\cos^2(\theta)\cdot\sin^2(\theta) \quad (5)$$

$$I(B_{1\mathrm{g}\parallel}) \propto d^2\cdot\sin^2(2\theta) \quad (6)$$

$$I(B_{1\mathrm{g}\perp}) \propto d^2\cdot\cos^2(2\theta) \quad (7)$$

$$I(B_{2\mathrm{g}\parallel}) = I(B_{2\mathrm{g}\perp}) = I(B_{3\mathrm{g}\parallel}) = I(B_{3\mathrm{g}\perp}) = 0 \quad (8)$$

The results reveal that when the laser is irradiated to the Bi_2Se_2S NWs (0 0 1) crystal plane, only the A_g mode and B_{1g} mode have Raman activity, which theoretical curves are displayed in Supplementary Fig. 7.

Supplementary Note 3

The entire linear polarization detection measurement system is shown in Supplementary Fig. 9. A modulated laser beam is changed into linearly polarized light through a Glan-Taylor prism (polarizer) and irradiate to the surface of the polarization-sensitive photodetector through the half-wave plate.

By rotating the half-wave plate, we can change the polarization direction of the linearly polarized light illuminated on the device. We define the angle between the vibration direction of linearly polarized light and the horizontal axis as the polarization angle. Since the vibration angle of the linearly polarized light compared to the original linearly polarized light changes twice the rotation angle of the half-wave plate. Therefore, when the half-wave plate is rotated by 10°, the vibration angle of the linearly polarized light will change by 20°.

Supplementary Note 4

Supplementary Fig. 14 displays the transfer characteristic curves of the transistor. From the result, we can see that the source-drain current (I_{ds}) decreases with the increase of the gate voltage (V_{gs}) and increases with the increase of the source-drain voltage (V_{ds}) at different V_{ds}, implying that the transistor is a typical p-type semiconductor, which threshold voltage (V_{th}) is about 1 V. The subthreshold swing around the Vth calculated by the formula $SS = dV_{gs}/d[\log(I_{ds})]$ is about 160 mV/decade, illustrating that the transistor can complete the transition from off-state to on-state within a small voltage range, which on/off current ratio is about 10^5 for V_{ds} = -0.5 V. To further characterize the response speed and stability of transistor during continuous switching, the frequency characteristics of Ids are measured. Supplementary Fig. 15a is the relationship between Ids and time when a 2 Hz square wave signal (V_{gs} = 1 V to 0 V) is applied to gate at V_{ds} = -0.5 V. The current is almost no jitter and

change in on-state of off-state, indicating excellent stability of the transistor. In addition, square wave voltage signal of multiple frequency including 10 Hz (Supplementary Fig. 15b) and 30 Hz (Supplementary Fig. 15c) are applied to gate, and the Ids corresponding to each frequency are extracted to obtain the frequency characteristic of the transistor shown in Supplementary Fig. 15d. Experimental results explain the operating frequency of the transistor can reach 30 Hz under the premise of keeping Ids stable. In general, all the parameters of the transistor can meet the requirements of the integrated system.

Supplementary Note 5

The structure of ANN was shown in Fig. 4a, including input layer, hidden layer and output layer. The number of neurons of three layers are 100, 100 and 6, respectively. In this work, the algorithm utilized for training and recognition processes is softmax linear classifier. Set learning rate to 0.0001 and during the training process, the value isn't changed. During the training process, images obtained via PSAS or obtained via Bi_2Se_2S based PS are delivered to the input layer.

The image dataset used to train and test neural network are generated based on the experimental results and MATLAB calculations. According to the fitting results of the Fig. 3c, we obtain the relationship formula between the polarized photocurrent and the polarization angle of the PSAS and Bi_2Se_2S based PS, respectively, formula 4 and formula 5:

$$I_{\mathrm{PASA}}(\delta) = 4.34 \times \sin^2(3.14 \times \delta/180 - 0.14) + 379 \times \cos^2(3.14 \times \delta/180 - 0.14) \quad (9)$$

$$I_{\mathrm{PS}}(\delta) = 6.2 \times \sin^2(3.14 \times \delta/180 - 0.21) + 8.19 \times \cos^2(3.14 \times \delta/180 - 0.21) \quad (10)$$

Each image can be viewed as three parts. First part is body patterns, including six kinds of letters (B, D, I, J, O, T). The value of each pixel is randomly generated by MATLAB. The random algorithm is composed based on the formula 9 and formula 10 and the random angle range is between 178° and 198°. The second part is background signals, which are also generated by random algorithm based on the above formulas and the random angle range is between 88° and 108°. As the third part, noise signal is also generated by random algorithm based on the above formulas and the random angle range is between 108° and 178°. Finally, we get two image datasets, which belong to PSAS and Bi_2Se_2S based PS (Supplementary Fig. 19). Supplementary Fig. 19a shows six types of images in two datasets. Supplementary Fig. 19b and c show images constructed according to the above method on the basis of six basic images, respectively. From Supplementary Fig. 19b and 19c, it can be seen that the image constructed by the PSAS, in which the background noise has little effect on the letter pattern. On the contrary, under the influence of background noise, the letters in the image constructed from PS shown in Supplementary Fig. 19b will be blurred a lot, which will cause the efficiency and accuracy of image recognition to decrease.

Supplementary Table 1. Polarized optoelectronic performance comparison of PS and PSAS.

Device	Wavelength	Power intensity (mW/cm²)	I_{py}	I_{px}	Anisotropic photocurrent ratio (I_{py}/I_{px})
PS	532 nm	35.873	872.0 pA	701.9 pA	1.24
	638 nm	111.368	819.5 pA	620.1 pA	1.32
	808 nm	53.980	724.6 pA	592.8 pA	1.22
PSAS	532 nm	37.714	7.393 nA	19.71 pA	375
	638 nm	113.579	37.89 nA	433.8 pA	87.3
	808 nm	77.443	5.411 nA	104.5 pA	51.77

Supplementary Table 2. Polarized optoelectronic performance comparison of PS based on low-dimensional nanostructure materials and our work.

Material	Growth method	Wavelength	Anisotropic photocurrent ratio (I_{py}/I_{px})	Digitization	Ref
$Sn^{II}Sn^{IV}S_3$	CVT	532 nm	1.3	None	1
SbI_3/Sb_2O_3	CVD	450 nm	3.14	None	2
Sb_2Se_3	CVD	532 nm	1.63	None	3
$CsPbBr_3$		513 nm	2.6	None	4
$CH_3NH_3PbI_3$		530 nm	1.3	None	5
ZnSb	CVD	1342 nm	1.28	None	6
SWNTs		980 nm	>2	None	7
ReS_2	CVT	517 nm	<3	None	8
GeAs		830 nm	4.4	None	9
$ReSe_2$	CVD	633 nm	~2	None	10
BP-on-WSe_2		1550 nm	5.9	None	11
CdSe	SLS	488 nm	1.3	None	12
Te	SSM	2300 nm	6	None	13
$GeSe_2$	CVD	405 nm	2.2	None	14
WS_2/Si	TDM	980 nm	2.8	None	15
GeS_2	MEM	UV	2.1	None	16
ZrS_3	CVD	520 nm	2.55	None	17
$BA_2CsPb_2Br_7$		408 nm	1.5	None	18
BAs-Si	MEM	2000 nm	1.2	None	19

**BP-MoS_2**	**MEM**	**3500 nm**	**100**	**None**	[20]
**p-BP/n-ReS_2**	**MEM**	**1064 nm**	**6.44**	**None**	[21]
**GeSe/MoS_2**	**MEM**	**532 nm**	**2.95**	**None**	[22]
**GeO_2-Ge**	**VLS**	**514 nm**	**4.26**	**None**	[23]
**Gr/$PdSe_2$/Ge**		**650 nm**	**112.2**	**None**	[24]
**BP/InSe**	**MEM**	**633 nm**	**10.76**	**None**	[25]
**β-$CsPbI_3$**		**530 nm**	**2.68**	**None**	[26]
**BP flakes**		**1200 nm**	**3.5**	**None**	[27]
**TiS_3/Si heterojunction**		**660 nm**	**2.62**	**None**	[28]
**Bi_2Se_2S based PS**	**CVD**	**532 nm**	**1.24**	**None**	**This work**
		**638 nm**	**1.32**	**None**	
**PSAS**		**532 nm**	**375**	**Yes**	**This work**
		**638 nm**	**87.3**		

Reference

1. Yang H*, et al.* Mixed-valence-driven quasi-1D $Sn^{II}Sn^{IV}S_3$ with highly polarization-sensitive UV-vis-NIR photoresponse. *Adv. Funct. Mater.* **29**, 1904416 (2019).
2. Xiao M*, et al.* Symmetry-reduction enhanced polarization-sensitive photodetection in core-shell SbI_3/Sb_2O_3 van der Waals heterostructure. *Small* **16**, 1907172 (2020).
3. Ma Z*, et al.* Chemical vapor deposition growth of high crystallinity Sb_2Se_3 nanowire with strong anisotropy for near-infrared photodetectors. *Small* **15**, 1805307 (2019).
4. Feng J*, et al.* Crystallographically aligned perovskite structures for high-performance polarization-sensitive photodetectors. *Adv. Mater.* **29**, 1605993 (2017).
5. Gao L*, et al.* Passivated single-crystalline $CH_3NH_3PbI_3$ nanowire photodetector with high detectivity and polarization sensitivity. *Nano Lett.* **16**, 7446-7454 (2016).
6. Chai R*, et al.* Non-layered ZnSb nanoplates for room temperature infrared polarized photodetectors. *J. Mater. Chem. C* **8**, 6388-6395 (2020).
7. Freitag M, Martin Y, Misewich JA, Martel R, Avouris PH. Photoconductivity of single carbon nanotubes. *Nano Lett.* **3**, 1067-1071 (2003).
8. Liu F*, et al.* Highly sensitive detection of polarized light using anisotropic

2D ReS2. *Adv. Funct. Mater.* **26**, 1169-1177 (2016).

9. Zhou Z*, et al.* Perpendicular optical reeversal of the linear dichroism and polarized photodetection in 2D GeAs. *ACS Nano* **12**, 12416-12423 (2018).
10. Zhang E*, et al.* Tunable ambipolar polarization-sensitive photodetectors based on high-anisotropy $ReSe_2$ nanosheets. *ACS Nano* **10**, 8067-8077 (2016).
11. Ye L*, et al.* Highly polarization sensitive infrared photodetector based on black phosphorus-on-WSe2 photogate vertical heterostructure. *Nano Energy* **37**, 53-60 (2017).
12. Singh A*, et al.* Polarization-sensitive nanowire photodetectors based on solution-synthesized CdSe quantum-wire solids. *Nano Lett.* **7**, 2999-3006 (2007).
13. Tong L*, et al.* Stable mid-infrared polarization imaging based on quasi-2D tellurium at room temperature. *Nat. Commun.* **11**, 2308 (2020).
14. Yan Y*, et al.* Direct wide bandgap 2D $GeSe_2$ monolayer toward anisotropic UV photodetection. *Adv. Opt. Mater.* **7**, 1900622 (2019).
15. Wu E*, et al.* In situ fabrication of 2D WS_2/Si type-II heterojunction for self-powered broadband photodetector with response up to mid-infrared. *ACS Photon* **6**, 565-572 (2019).
16. Yang Y*, et al.* Polarization-sensitive ultraviolet photodetection of anisotropic 2D GeS_2. *Adv. Funct. Mater.* **29**, 1900411 (2019).
17. Wang X*, et al.* Highly polarized photoelectrical response in vdW ZrS_3 nanoribbons. *Adv. Electron. Mater.* **5**, 1900419 (2019).
18. Wang J*, et al.* Ultrasensitive polarized-light photodetectors based on 2D hybrid perovskite ferroelectric crystals with a low detection limit. *Sci. Bull.* **66**, 158-163 (2021).
19. Nidhi, Jakhar A, Uddin W, Kumar J, Nautiyal T, Das S. Nanolayered black arsenic-silicon lateral heterojunction photodetector for visible to mid-infrared wavelengths. *ACS. Appl. Nano Mater.* **3**, 9401-9409 (2020).
20. Bullock J*, et al.* Polarization-resolved black phosphorus/molybdenum disulfide mid-wave infrared photodiodes with high detectivity at room temperature. *Nat. Photon.* **12**, 601-607 (2018).
21. Zhu W, Wei X, Yan F, Lv Q, Hu C, Wang K. Broadband polarized photodetector based on p-BP/n-ReS_2 heterojunction. *J. Semicond.* **40**, 092001 (2019).
22. Xin Y*, et al.* Polarization-sensitive self-powered type-II GeSe/MoS_2 van der waals heterojunction photodetector. *ACS. Appl. Mater. Interfaces* **12**, 15406-15413 (2020).
23. Mukherjee S, Das K, Das S, Ray SK. Highly responsive, polarization sensitive, self-biased single GeO_2-Ge nanowire device for broadband and low power photodetectors. *ACS. Photon* **5**, 4170-4178 (2018).
24. Wu D*, et al.* Highly polarization-sensitive, broadband, self-powered photodetector based on graphene/$PdSe_2$/germanium heterojunction.

ACS Nano **13**, 9907-9917 (2019).

25. Zhao S*, et al.* Highly polarized and fast photoresponse of black phosphorus-InSe vertical p-n heterojunctions. *Adv. Funct. Mater.* **28**, 1802011 (2018).
26. Zhou Y*, et al.* Flexible linearly polarized photodetectors based on all-inorganic perovskite $CsPbI_3$ nanowires. *Adv. Opt. Mater.* **6**, 1800679 (2018).
27. Yuan H*, et al.* Polarization-sensitive broadband photodetector using a black phosphorus vertical p-n junction. *Nat. Nanotechnol.* **10**, 707-713 (2015).
28. Niu Y*, et al.* Polarization-sensitive and broadband photodetection based on a mixed-dimensionality TiS_3/Si p-n junction. *Adv. Opt. Mater.* **6**, 1800351 (2018).
